# Characterization of a New Sesquiterpene and Antifungal Activities of Chemical Constituents from *Dryopteris fragrans* (L.) Schott

**DOI:** 10.3390/molecules19010507

**Published:** 2014-01-02

**Authors:** Yu-Hong Huang, Wei-Min Zeng, Guo-Yu Li, Guo-Qing Liu, Dan-Dan Zhao, Jing Wang, Yan-Long Zhang

**Affiliations:** 1 Key Laboratory of Molecular Biology of Heilongjiang Province, College of Life Science, Heilongiang University, Harbin 150080, China; E-Mails: YHHuanghd@163.com (Y.-H.H.); wmzenghd@163.com (W.-M.Z.); waterpowerful@sina.com (G.-Q.L.); ddzhaohd@163.com (D.-D.Z.); ebenbenebenben@sina.com (J.W.); 2 R&D Center, Harbin Pharmaceutical Group, Harbin 150060, China; 3 Pharmaceutical College, Harbin Medical University, Harbin 150086, China; E-Mail: leegy@163.com

**Keywords:** *Dryopteris fragrans* (L.) Schott, chemical constituents, activity screen, antifungal activity

## Abstract

One new sesquiterpene and six known compounds were isolated from *Dryopteris fragrans* (L.) Schot. They were identified as 3-*O*-*β*-D-glucopyranosylalbicanol-11-*O*-*β*-D-glucopyranoside (**1**), dihydroconiferylalcohol (**2**), (*E*)-3-(4-hydroxyphenyl)acrylic acid (**3**), esculetin (**4**), 5,7-dihydroxy-2-hydroxymethylchromone (**5**), eriodictyol (**6**) and isoorientin (**7**) by UV, MS, 1D-NMR and 2D-NMR spectroscopy. The antifungal activities of the seven isolated compounds were screened. Compounds **2**, **3**, **4** and **5** showed obvious activities against *Microsporum canis* and *Epidermophyton floccosum*.

## 1. Introduction

*Dryopteris fragrans* (L.) Schott, a deciduous perennial herb from the genus *Dryopteris* (Dryopteridaceae), is mainly distributed in Northeast China, Russia, Japan, Korea and North America. The herb is used for treatment of skin diseases such as psoriasis, rashes, dermatitis, other skin diseases, barbiers and arthritis [1,2,3,4]. Previous research had discovered phloroglucins, terpenes, flavonoids, saponins, essential oils and sterols in this plant, and activity screenings of the related constituents have become popular [2,5,6]. Our research group has reported one new phenolic acid from the herb [7,8]. In this paper, we report the isolation and structural identification of one new sesquiterpene together with six known compounds which were obtained from genus *Dryopteris* for the first time and the assay of their antifungal activity in order to identify the active compounds.

## 2. Results and Discussion

### 2.1. Chemical Structure Identification and Spectroscopic Data

Compound **1**, obtained as a light yellow oil, had a molecular formula of C_27_H_46_O_12_ based on the HRESIMS ([M+Na]^+^ 585.2890), which indicated five degrees of unsaturation. The UV_max_ absorption at 205.242 nm indicated an isolated chromophore in the structure. Four methyl groups (*δ*_H_ 0.80, 1.17, 1.26, 2.05), one olefinic proton (*δ*_H_ 5.47), one oxygenated methine proton (*δ*_H_ 4.06) and two oxygenated methylenes (*δ*_H_ 4.01) were observed. Furthermore, we deduced the presence of two sugar residues from the signal of two anomeric protons at *δ*_H_ 4.84 (1H, d, *J* = 7.8 Hz) and *δ*_H_ 4.90 (1H, d, *J* = 7.8 Hz, Table 1). The acid hydrolysis of **1** with aqueous 2 M HCl yielded D-glucose, which was identified by GC comparison with a sugar standard.

**Table 1:** ^1^H- and ^13^C-NMR data of compound **1**.

No.	H	C	No.	H	C
1	2.18 (1H, m), 1.82 (1H, m)	37.8	15	1.26 (3H, s)	28.2
2		24.6	1'	4.90 (1H, d, *J* = 7.8 Hz)	106.9
3	4.06 (1H, m)	89.0	2'	4.01 (m)	75.8
4		39.4	3'	4.25 (m)	78.8
5	1.91 (1H, brs)	50.1	4'	4.27 (m)	71.8
6	2.18 (1H, m), 1.82 (1H, m)	27.9	5'	4.29 (m)	78.4
7	5.47 (1H, brs)	122.7	6'	4.61 (1H, dd, *J* = 12.0, 1.2 Hz) 3.41 (1H, dd, *J* = 12.0, 3.2 Hz)	63.1
8		134.4	1''	4.84 (1H, d, *J* = 7.8 Hz)	105.3
9		54.9	2''	4.01 (m)	75.3
10		35.6	3''	4.25 (m)	78.7
11	4.01 (1H, m)	69.9	4''	4.27 (m)	71.8
12	2.05 (3H, s)	22.5	5''	4.29 (m)	78.7
13	0.80 (3H, s)	14.8	6''	4.61 (1H, dd, *J* = 12.0, 1.2 Hz) 3.63 (1H, dd, *J* = 12.0, 6.6 Hz)	62.9
14	1.17 (3H, s)	16.6			

In the ^13^C-NMR spectrum (Table 1) 27 carbon signals were resolved. Besides the carbon signals of the two D-glucoses there were also 15 carbon signals comprising four methyls (*δ*_C_ 22.5, 14.8, 16.6, 28.2), four methylenes (*δ*_C_ 37.8, 27.9, 24.6, 69.9), four methines (*δ*_C_ 89.0, 50.1, 122.7, 54.9), and three quaternary carbons (*δ*_C_ 39.4, 134.4, 35.6) as classified by their chemical shifts and from the HSQC spectrum. All of the signals above suggested the aglycone of compound **1** was a sesquiterpene.

**Figure 1:**
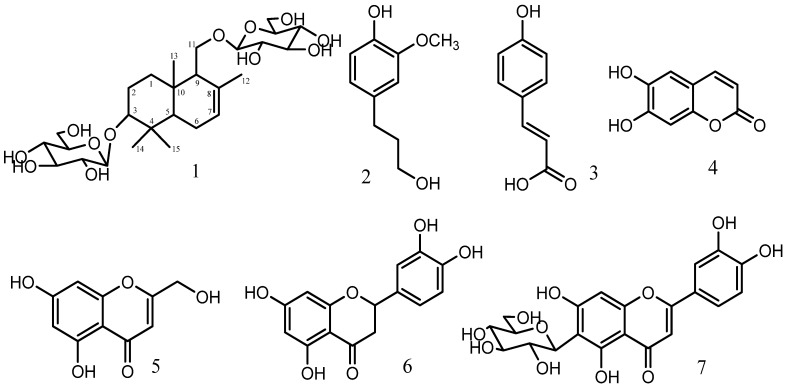
Chemical strctures of compounds **1**–**7**.

The two sugar residues in compound **1** were linked at C-3 and C-11, as determined by the HMBC correlations from δ_H_ 4.90 (H-1') to δ_C_ 89.0 (C-3) and from δ_H_ 4.84 (H-1'') to δ_C_ 69.9 (C-11). Furthermore HMBC correlations between δ_H_ 1.17 (H-14) and 1.25 (H-15) and δ_C_ 39.4 (C-4), between δ_H_ 2.05 (H-12) and δ_C_ 134.4 (C-8) and between δ_H_ 0.80 (H-11) and δ_C_ 35.6 (C-10), suggested four methyl groups were attached to C-4, C-8 and C-10 respectively. Therefore, the structure of compound **1** was established as shown in Figure 1. The known compounds **2**–**7** were identified by comparison of the spectral data (^1^H-NMR, ^13^C-NMR) with the literature data.

**Table 2:** Minimum inhibitory concentration (MIC) distribution of the seven isolated compounds against *M. canis* and *E. floccosum.*

Compounds No.	Minimum Inhibitory Concentration (MIC Values, μg/mL)	
	*Microsporum canis*	*Epidermophyton floccosum*
**1**	na	na
**2**	0.0625	<0.015625
**3**	<0.015625	0.03125
**4**	<0.015625	<0.015625
**5**	<0.015625	0.03125
**6**	8	4
**7**	>32	0.5
Griseofulvin	1	0.03125

na = inactive.

### 2.2. Screening for *In Vitro* Antifungal Activities [9,10,11]

Compounds **1**–**7** were screened for antifungal acitvities against *Microsporum canis* and *Epidermophyton floccosum*. The corresponding Minimum Inhibitory Concentration (MIC, μg/mL) values are listed in Table 2. Compounds **2**–**5** showed big differences compared with the reference standard griseofulvin (MIC value 1.0 μg/mL–0.03125 μg/mL. Table 2). The new compound **1** was inactive.

## 3. Experimental

### 3.1. General

^1^H- and ^13^C-NMR spectra were recorded on a Bruker AM-400 (Bruker Corporation, Fällanden, Switzerland) with TMS as an internal standard. ESIMS were recorded on API QSTAR Pulsari (Applied Biosystems, MDS Sciex, Framingham, MA, USA) and VG-Autospec-3000 mass spectrometers (AB SCIEX mass spectrometers, Framingham, MA, USA). UV spectra were obtained on a Shimadzu UV-2401PC spectrophotometer (Shimadzu, Kyoto, Japan). Optical rotations were measured on a SEPA-300 polarimeter (Horiba, Kyoto, Japan). The GC was performed on HP6890 N gas chromatograph (Agilent, Milton-Freewater, OR, USA) equipped with a flame ionization detector and a HP-5 capillary column (30 m × 0.32 mm × 0.25 μm), injector temperature: 230 °C, detector temperature: 250 °C, column temperature ramp: 150–280 °C at a rate of 5 °C/min. Silica gel (100–200 and 200–300 mesh, Qingdao Marine Chemical Co. Ltd., Qingdao, China), AB-8 Macroporous adsorption resin (Nankai Chemical Co. Ltd., Tianjin, China), MCI gel (75–150 μm (Mitsubishi Chemical Corporation, Kyoto, Japan)), and Sephadex LH-20 (GE Healthcare Bio-Sciences AB, Uppsala, Sweden) were used for column chromatography (CC) Semi-preparative HPLC was performed on an Agilent 1100 liquid chromatography (Agilent Corporation, Waldbronn, Germany) with a Zorbax SB-C18 (9.4 mm × 25 cm) column (Agilent Corporation). Silica gel GF254 (Qingdao Marine Chemical Inc.) were employed for thin-layer chromatography (TLC).

### 3.2. Plant Material

The whole plant of *Dryopteris fragrans* (*L.*) Schott were collected in Wu-da-lian-chi, Heilongjiang Province, China in August 2009, and identified by Prof. Zhen-Yue Wang, Heilongjiang University of Chinese Medicine. The voucher specimen (registration number: XLMJ-20110812) of this plant was deposited in the Herbarium of Heilongjiang University of Chinese Medicine, Harbin, China.

### 3.3. Extraction and Isolation

Air-dried, powdered whole plants of *D. fragrans* (L.) Schott (20 kg) were extracted three times at room temperature for 2.0 h with 95% ethanol (200 L, 160 L and 120 L). The combined 95% EtOH extracts were evaporated to near dryness and the dry residue (1.0 kg) was suspended in H_2_O and successively eluted from an AB-8 macroporous adsorption resin column with H_2_O (3 × 4 L), 30% EtOH (3 × 5.0 L), 60% EtOH (3 × 5.0 L) and 95% EtOH (3 × 5.0 L). The 30% EtOH fraction (100 g) was subjected to silica gel column chromatography with a CHCl_3_/MeOH (100:0→1:1, v/v) gradient as eluent to give fractions D_1_→D_5_. Repeated silica gel chromatography of fraction D_2_ (20 g) eluting with CHCl_3_/MeOH (30:0→10:1, v/v) yielded compounds **3** (75 mg) and **5** (135 mg). Compounds **2** (55 mg) and **4** (5.6 mg) were isolated from D_1_ (10 g) by silica gel column chromatography elutin g with CHCl_3_. D_3_ (3.0 g) was subjected to ODS column chromatography with MeOH/H_2_O (35:65, v/v) as eluent to yield compound **6**, D_4_ (3.5g) was purified by preparative HPLC on a ODS column (10 μm, 20 × 300 mm, flow rate 8 mL/min) with MeOH/H_2_O (35:65) and MeOH/H_2_O (45:55) as eluents to give **1** (65 mg), and **7** (95 mg).

### 3.4. Characterization of Isolated Compounds

*3-O-β-D-Glucopyranosylalbicanol-11-O-β-D-glucopyranoside* (**1**). A light yellow oil. ^1^H-NMR (MeOD) *δ*_H_: 5.47 (brs, 1H, H-7), 1.91 (brs, 1H, H-5), 2.05 (s, 3H, -CH_3_), 1.26 (s, 3H, -CH_3_), 1.17 (s, 3H, -CH_3_), 0.80 (s, 3H, -CH_3_), 4.90 (d, *J* = 7.8 Hz, 1H, H-1'), 4.84 (d, *J* = 7.8 Hz, 1H, H-1''). ^13^C-NMR (MeOD) *δ*_C_: 37.8 (C-1), 24.6 (C-2), 89.0 (C-3), 39.4 (C-4), 50.1 (C-5), 27.9 (C-6), 122.7 (C-7), 134.4 (C-8), 54.9 (C-9), 35.6 (C-10), 69.9 (C-11), 22.5 (C-12), 14.9 (C-13), 16.6 (C-14), 28.2 (C-15), 106.9 (C-1'), 75.8 (C-2'), 78.8 (C-3'), 71.8 (C-4'), 78.4 (C-5'), 63.1 (C-6'), 105.3 (C-1''), 75.3 (C-2''), 78.7 (C-3''), 71.8 (C-4''), 78.7 (C-5''), 62.9 (C-6'').

*Dihydroconiferylalcohol* (**2**). Colorless crystals. ^1^H-NMR (MeOD) *δ*_H_: 7.28 (s, 1H), 6.77 (d, *J* = 1.8 Hz, 1H, H-2), 6.70 (d, *J* = 8.0 Hz, 1H, H-5), 6.62 (dd, *J* = 8.0, 1.8 Hz, 1H, H-6), 2.59 (t, *J* = 7.6 Hz, 2H, H-7), 1.80 (m, 2H, H-8), 3.56 (t, *J* = 6.5 Hz, 2H, H-9), 3.82 (s, 3H, -OCH_3_). ^13^C-NMR (MeOD) *δ*_C_: 134.9 (s, C-1), 113.1 (d, C-2), 148.8 (s, C-3), 145.5 (s, C-4), 116.1 (d, C-5), 121.8 (d, C-6), 32.7 (t, C-7), 35.7 (t, C-8), 62.3 (t, C-9), 56.3 (q, C-10).

*(E)-3-(4-Hydroxyphenyl)acrylic acid* (**3**). Pale yellow powder. ^1^H-NMR (acetone*-d_6_*) *δ*_H_: 7.54 (d, *J* = 8.6 Hz, 2H, H-2, H-6), 6.89 (d, *J* = 8.6 Hz, 2H, H-3, H-5), 7.63 (d, *J* = 16.0 Hz, 1H, H-7), 6.35 (d, *J* = 16.0 Hz, 1H, H-8). ^13^C-NMR (acetone*-d_6_*) *δ*: 127.2 (s, C-1), 131.3 (d, C-2, C-6), 116.2 (d, C-3, C-5), 161.1 (s, C-4), 146.1 (d, C-7), 117.1 (d, C-8), 169.9 (s, C-9).

*Esculetin* (**4**). Green amorphous powder. ^1^H-NMR (MeOD) *δ*_H_: 6.18 (d, *J* = 9.0 Hz, 1H, H-3), 7.79 (d, *J* = 9.0 Hz, 1H, H-4), 6.94 (s, 1H, H-5), 6.75 (brs, 1H, H-8). ^13^C-NMR (MeOD) *δ*_C_: 164.3 (s, C-2), 112.8 (d, C-3), 146.1 (d, C-4), 113.0 (d, C-5), 144.6 (s, C-6), 150.5 (s, C-7), 103.6 (d, C-8), 152.0 (s, C-9), 112.5 (s, C-10).

*5,7-Dihydroxy-2-hydroxymethylchromone* (**5**). Pale yellow crystals. ^1^H-NMR (MeOD) *δ*_H_: 6.22 (s, H, H-3), 6.27 (brs, 1H, H-6), 6.34 (brs, 1H, H-8), 4.52 (s, 2H, H-11). ^13^C-NMR (MeOD) δ_C_: 164.3 (s, C-2), 112.8 (d, C-3), 146.1 (d, C-4), 113.0 (d, C-5), 144.6 (s, C-6), 150.5 (s, C-7), 103.6 (d, C-8), 152.0 (s, C-9), 112.5 (s, C-10).

*Eriodictyol* (**6**). Pale yellow crystals. ^1^H-NMR (MeOD) *δ*_H_: 5.36 (dd, *J* = 2.9, 12.9 Hz, 1H, H-2), 2.69 (dd, *J* = 2.9, 17.4 Hz, 2H, H-3α, H-3β), 5.91 (d, *J* = 2.1 Hz, 1H, H-6), 5.93 (d, *J* = 2.1 Hz, 1H, H-8), 7.01 (s, 1H, H-2'), 6.84 (s, 2H, H-5', H-6'). ^13^C-NMR (MeOD) *δ*_C_: 79.9 (d, C-2), 43.4 (t, C-3), 197.3 (s, C-4), 165.1 (s, C-5), 96.7 (d, C-6), 167.7 (s, C-7), 95.8 (d, C-8), 164.8 (s, C-9), 103.8 (s, C-10), 131.2 (s, C-1'), 114.6 (d, C-2'), 146.1 (s, C-3'), 146.5 (s, C-4'), 115.9 (d, C-5'), 119.0 (d, C-6').

*Isoorientin* (**7**). Pale yellow powder. ^1^H-NMR (MeOD) *δ*_H_: 13.96 (s, 1H, 5-OH), 6.73 (s, 1H, H-3), 6.77 (s, 1H, H-8), 7.56 (brs, 1H, H-2'), 7.29 (d, *J* = 8.5 Hz, 1H, H-5'), 7.85 (d, *J* = 8.5 Hz, 1H, H-6'), 5.97(d, *J* = 9.8 Hz, 1H, H-1''), 4.23–5.10 (m, 5H, H-2'', H-3'', H-4'', H-5'', H-6''). ^13^C-NMR (MeOD) *δ*_C_: 165.1 (s, C-2), 103.4 (d, C-3), 183.1 (s, C-4), 157.4 (s, C-5), 106.1 (s, C-6), 164.6 (s, C-7), 99.2 (d, C-8), 162.3 (s, C-9), 105.4 (s, C-10), 123.4 (s, C-1'), 115.8 (d, C-2'), 147.6 (s, C-3'), 151.6 (s, C-4'), 117.1 (d, C-5'), 120.3 (d, C-6'), 75.8 (d, C-1''), 72.3 (d, C-2''), 81.1 (d, C-3''), 73.1 (d, C-4''), 83.6 (d, C-5''), 63.1 (t, C-6'').

### 3.5. Acid Hydrolysis

Compound **1** (5 mg) was hydrolyzed with 2 mol/L HCl (5 mL) for 5 h at 90 °C. After cooling to room temperature, the reaction mixture was extracted with EtOAc (5 mL) three times. Each remaining aqueous layer was neutralized with 0.5 N NaOH and then freeze-dried to give a residue. The residue was dissolved in pyridine (2 mL) and L-cysteine methyl ester hydrochloride (3 mg) was added to the solution. The solution was kept at 60 °C for 1 h. Then trimethylchlorosilane (0.5 mL) was added to the reaction mixture and heated at 60 °C for another 30 min. After centrifugation, the supernatant was analyzed by GC. The sugar derivatives obtained from compounds **1** showed a single peak at 32.3 min. The retention time was similar to that of a D-glucose derivative, so the sugar was identified as D-glucose.

### 3.6. Microsporum Canis and Epidermophyton floccosum Strains [8,9,10]

*M. canis* and *E. floccosum* were obtained from the fungus preservation center in the China Academy of Sciences and the Institute of Medicine of Dermatology, respectively. Antifungal tests were performed using the method of Dilution Antifungal Susceptibility Testing of Filamentous Fungi as described by the National Committee for Clinical Laboratory Standards [9].

## 4. Conclusions

A new sesquiterpene **1** and known compounds **2**–**7** were isolated from the genus *Dryopteris* for the first time. The antifungal activity screening results with *Microsporum canis* and *Epidermophyton floccosum* showed that compounds **2**, **3**, **4** and **5** have remarkable activities against both species.
